# A226V Strains of Chikungunya Virus, Réunion Island, 2010

**DOI:** 10.3201/eid1702.101056

**Published:** 2011-02

**Authors:** Eric D’Ortenzio, Marc Grandadam, Elsa Balleydier, Marie-Christine Jaffar-Bandjee, Alain Michault, Elise Brottet, Marie Baville, Laurent Filleul

**Affiliations:** Author affiliations: Regional Office of French Institute for Public Health Surveillance, Saint-Denis, Réunion Island (E. D’Ortenzio, E. Balleydier, E. Brottet, L. Filleul);; Institut Pasteur, Paris, France (M. Grandadam);; Regional Hospital, Saint-Denis (M.-C. Jaffar-Bandjee);; Regional Hospital, Saint-Pierre, Réunion Island (A. Michault);; Health Agency of Indian Ocean, Saint-Denis (M. Baville)

**Keywords:** Chikungunya virus, disease outbreaks, Indian Ocean, reemergence, Réunion Island, arboviruses, viruses, vector-borne infections, letter

**To the Editor:** Chikungunya virus (CHIKV) first emerged in Indian Ocean islands off the eastern coast of Africa in 2005 and was responsible for large-scale epidemics on the islands of Réunion, Comoros, Mayotte, Mauritius, Madagascar, and Seychelles (*1*–*4*). On Réunion Island, a French overseas territory of 810,000 inhabitants, herd immunity reached 38% in October 2006 (*5*). Molecular epidemiology of the strain responsible for these outbreaks indicated that it had originated in Kenya (*6*). The epidemic on Réunion Island was associated with a mutation in the envelope protein gene (E1-A226V) that improves replication and transmission efficiency in *Aedes albopictus* mosquitoes (*7*).

Since 2006, the Regional Office of the French Institute for Public Health Surveillance in the Indian Ocean has conducted epidemiologic and biological surveillance for CHIKV infection. Case definitions have been described (*8*). During December 2006–July 2009, no confirmed case was detected on Réunion Island and Mayotte, but new outbreaks were reported in Madagascar (*9*). In August 2009, a cluster of cases was identified on the western coast of Réunion Island (*8*).

We report an outbreak of CHIKV infection that occurred on Réunion Island in 2010. The first case was detected on March 17, 2010. As of July 6, a total of 100 confirmed and 32 probable cases had been identified (Figure A1). Median age of case-patients was 39 years (range 6 months–80 years), and the ratio of male to female case-patients was 0.81:1. In addition to fever (95%), case-patients had arthralgia (95%), headache (78%), and myalgia (75%). Seven (5%) were admitted to hospitals. No severe illness or death was reported. The outbreak remained largely restricted to residents of Saint Paul (75%) on the western coast. Sporadic cases in other cities also were detected.

Sequence comparison based on partial envelope gene or complete genome showed a high level (>99.6%) of nucleotide and amino acid identity of 2010 isolates from Réunion Island with the strains of the 2009 sporadic cases on Réunion Island, as well as with the Malagasy strains circulating since 2006. All isolates sequenced bore the A226V substitution within the E1 protein. Altogether, these results support the hypothesis of a continuous circulation of A226V strains in the southwestern Indian Ocean since 2006 and the possible reintroduction of CHIKV on Réunion Island, most probably from Madagascar. Once again, human travel may have contributed to the rapid spread of the virus between islands because imported and autochthonous cases on Réunion Island occurred after a holiday period for residents on Réunion Island who often traveled to Madagascar. Migration and birth rate on Réunion Island might have contributed to a decrease in the immunity of the population. Furthermore, seroprevalence in 2007 was not homogenous throughout the territory. A hypothesis would be that a lower immunity of the population in the Saint Paul area and environmental and vectorial characteristics contributed to the emergence of this CHIKV disease cluster.

On Réunion Island, *Ae. albopictus* mosquitoes have been described as the main vector responsible for transmitting CHIKV (*10*). The austral winter may contribute to moderate vector activity and transmission. We cannot exclude a continuous transmission until next austral summer, followed by an increase of cases and an extension to the whole island, as occurred in 2005 (*1*). Epidemiologic and entomologic surveillance has been reorganized to prevent this risk. Medical staff, the general population, and travelers have been informed about the situation through the news media and meetings organized by health authorities, and recommendations have been issued about destroying mosquito breeding sites and preventing mosquito bites.

In recent years, the area of circulation and the epidemic potential of CHIKV have increased, and CHIKV has emerged as a major public health problem. This outbreak could be a new warning to Réunion Island health authorities about the need for preparation not only for CHIKV but also for dengue virus (DENV). With the extent of human travel to and from areas with active CHIKV and DENV circulation, viremic returning travelers constitute an ongoing risk for introduction of such viruses on Réunion Island. In May 2010, two locally acquired DENV-3 cases were also detected, illustrating this threat. These cases occurred during an outbreak of DENV-3 in Comoros Island. Public health efforts to control *Ae. albopictus* mosquitoes have not been completely effective.

This outbreak of CHIKV infection, the detection of autochthonous cases of DENV infection, and the influenza season on Réunion Island emphasize the difficulty of making the appropriate clinical diagnosis. Clinicians and biologists should be aware of the cocirculation of CHIKV, DENV, and influenza viruses. The reemergence of CHIKV on Réunion Island illustrates the permanent threat of circulation of exotic pathogens in the Indian Ocean and the need for strong epidemiologic and laboratory surveillance. Human travel and the geographic expansion of *Ae. albopictus* mosquitoes raise concern for the spread of CHIKV in Europe and North America.

## Figures and Tables

**Figure A1:**
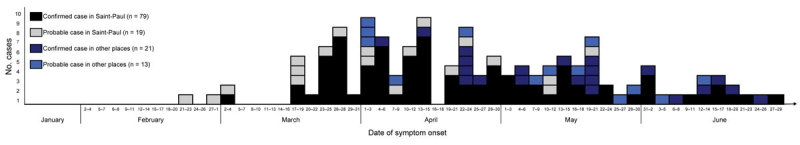
Cases of chikungunya infection, Réunion Island, 2010
